# Effects of biomechanical forces on the biological behavior of cancer stem cells

**DOI:** 10.7150/jca.60893

**Published:** 2021-08-08

**Authors:** Bo Ren Tian, Wei Fan Lin, Yan Zhang

**Affiliations:** MOE Key Laboratory of Gene Function and Regulation, School of Life Sciences, Sun Yat-sen University, Guangzhou 510006, Guangdong, People's Republic of China.

**Keywords:** tumor microenvironment, cancer stem cell, matrix stiffness, shear stress, compression and tension

## Abstract

Cancer stem cells (CSCs), dynamic subsets of cancer cells, are responsible for malignant progression. The unique properties of CSCs, including self-renewal, differentiation, and malignancy, closely depend on the tumor microenvironment. Mechanical components in the microenvironment, including matrix stiffness, fluid shear stress, compression and tension stress, affect the fate of CSCs and further influence the cancer process. This paper reviews recent studies of mechanical components and CSCs, and further discusses the intrinsic correlation among them. Regulatory mechanisms of mechanical microenvironment, which act on CSCs, have great potential for clinical application and provide different perspectives to drugs and treatment design.

## Introduction

Cancer is the second leading cause of death worldwide and poses a great threat to human life and health. A variety of treatments, including surgery, chemotherapy, radiation therapy, and molecule-targeted therapies, such as angiogenesis inhibitors, tyrosine kinase inhibitors, and monoclonal antibodies, have been widely deployed against cancer, yet the outcomes for patients, particularly those with the most aggressive cancers, remain mixed 1, 2. Although many studies have been conducted on the tumorigenesis and metastasis of cancer, there are still various aspects not well understood.

Within cancer tissues, there are several dynamic subsets of cancer cells considered to be cancer stem cells (CSCs) or stem cell-like cancer cells 3, 4. CSCs are often identified with abundant expression of stem cell markers, long-term clonal proliferation, tumoriginecity, facilitating metastasis and resistance to chemotherapy. Since the first demonstration of the presence of CSCs in leukemia 5, the existence of CSCs has been successfully demonstrated in a variety of cancers 6-10. Both clinical and experimental data indicate that CSCs can survive during chemotherapy and radiation therapy 11. The existence of CSCs is a barrier that limits therapeutic effect 12, although CSCs may be dormant and quiescent for long periods of time 13. Thorough studies on CSCs may offer theoretical guidance for cancer therapy in clinical practice.

Many studies have demonstrated that the microenvironment could regulate stem cell fate by providing biological conditions, including those that are cell-to-cell, cell- or non-cell factors, and mechanical stimuli 14-17, which showed that mechanical factors could affect stem cell proliferation, migration, differentiation, stemness maintenance, and other biological behaviors 18, 19. These mechanical components include matrix stiffness, fluid shear stress, cyclic stretching or compression, even gravity environments, or topological structures 20-22.

In recent years, the biomechanical microenvironment of cancer has attracted more attention 23. Several studies have shown that the biomechanical microenvironment in solid tumors differs completely from that in surrounding tissues 24. Throughout the process of cancer development, excessive and disordered cell proliferation will lead to abnormal development of the biomechanical microenvironment, including solid stress, increased tissue stiffness, and an abnormal interstitial fluid pressure (IFP) 25. This special biomechanical microenvironment might play an important role in maintaining the survival of CSCs and cancer-associated cells 26. For example, in the process of hematogenous metastasis, cancer cells leave the primary niche and enter the circulatory system with a unique biomechanical microenvironment. During the transition through the circulatory system, cancer cells are subjected to hemodynamic forces, immunological stress, and collisions with host cells, such as blood cells and the endothelial cells lining the vessel wall 27. These biomechanical stressors could affect cancer cell fate and the ability to establish metastatic foci 28. Moreover, all of these biomechanical stressors could affect CSCs stemness during cancer progress (Table 1).

## The biomechanical microenvironment in solid tumor

### Matrix stiffness

The biomechanical microenvironment in cancer tissue has three unique characteristics: solid stress caused by unregulated proliferation of cancer cells, increased extracellular matrix (ECM) stiffness, and abnormal IFP 26. The increasing accumulation of ECM changes tissue density, which eventually leads to a gradual change in the tissue's stiffness 29. It is generally believed that the stiffness of solid tumor is much higher than that of normal tissue 30. For example, a normal mammary gland has a modulus of elasticity of less than 200 Pa, while the tumor over 4 kPa; normal liver tissue has a stiffness between 4 and 10 kPa, yet the stiffness of a liver cancer tends to be 20 to 50 kPa or higher 29, 31. As osteosarcomas arise in the hardest tissue of the body, the dogma stating that solid tumors are macroscopically harder than the healthy tissue from which they originate does not apply in this case 32. It was shown that osteosarcomas are markedly weaker and softer, exhibiting a step-by-step bone matrix reduction and degradation, indicative of sequential bone structural failures 33. There are also differences in stiffness within tumors. In the interior of a solid tumor, different areas show distinct stiffness profiles, and the stiffness in the invasion frontier is much stiffer than that of the central area 34.

### Shear stress

**Figure 1:**
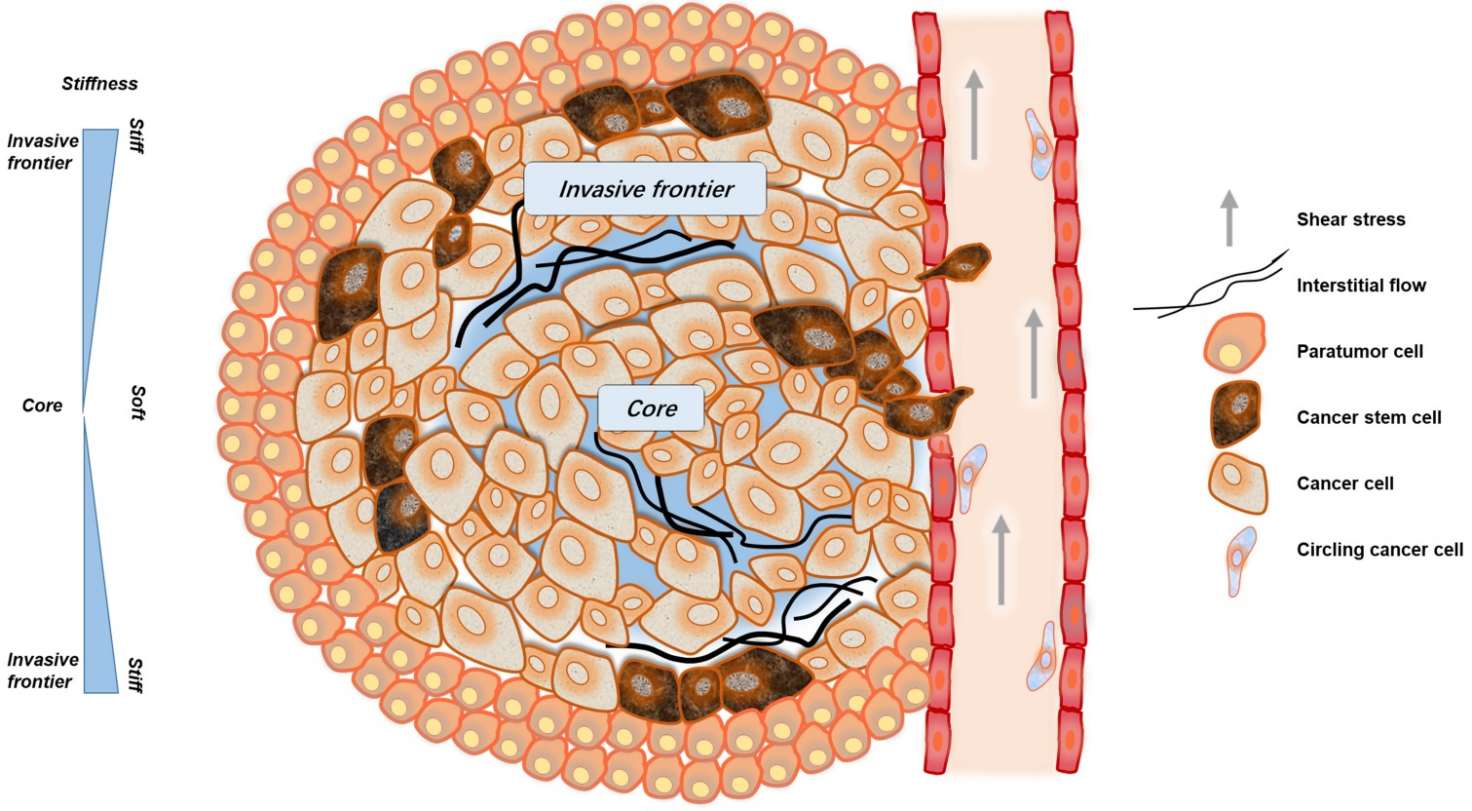
The mechanical microenvironment of cancer. CSCs are not uniformly distributed in cancer tissues. More CSCs are distributed in invasive frontiers to facilitate malignant metastasis. At the invasive frontier, CSCs are subjected to the forces of increased matrix stiffness, interstitial fluid pressure, and the tensile force of surrounding tissues. When CSCs enter the blood vessels, they are subjected to fluid shear forces generated by blood flow. These mechanical factors play important roles in maintaining the characteristics of CSCs.

When cancer tissues enlarge to a certain size, they tend to be hypoxic. This could cause cancer cells or cancer-related cells to produce abnormal secretion of vascular endothelial growth factor (VEGF) or other angiogenic factors, causing disordered angiogenesis and lymphangiogenesis 35. However, these neo-vessels are poorly functional, with irregular networks and high permeability 28. Therefore, liquids and macromolecules are more likely to infiltrate the interspace, breaking the balance of osmotic pressure, resulting in elevated tumor IFP 36. In solid tumors, the increasing number of lymphatic vessels around the tumor which is coupled with high IFP, leads to a rise in tumor fluid flux 37. The IFP within solid tumors, although slower in flow rate compared with blood shear stress, still produces a certain fluid shear stress that can influence cell behavior (Fig. 1). In addition to the fluid shearing force, the flow of the interstitial fluid causes the concentration gradient of the solute molecules or signal molecules, and the concentration gradient is generated inside or outside the cell, contributing to signal transmission and the exchange of substances 26.

In the circulatory system, cancer cells are subjected to fluid shear stress generated by blood flow, the fluid shear stress generated by the circulatory system is much greater than that generated by interstitial flow 38, 39. Only a small fraction of cancer cells, CSCs or circulating tumor cells (CTCs), can overcome or even exploit the effects of fluid shear, gradually escape the circulation and successfully arise metastatic tumors 40, 41 (Fig. 1).

### Compression and tension stress

Solid stress is always present in solid cancer tissues. Owing to the epithelial frontier, all unregulated cancer cells grow in a limited space, which inevitably generates more solid stress in tumors compared with surrounding tissues 26. The solid pressure within tumor reaches 45-120 mm Hg, so that exceeding the lymphatic or blood vessels pressure (6-17 mm Hg) can cause the collapse of the tumor blood vessels 42.

**Figure 2:**
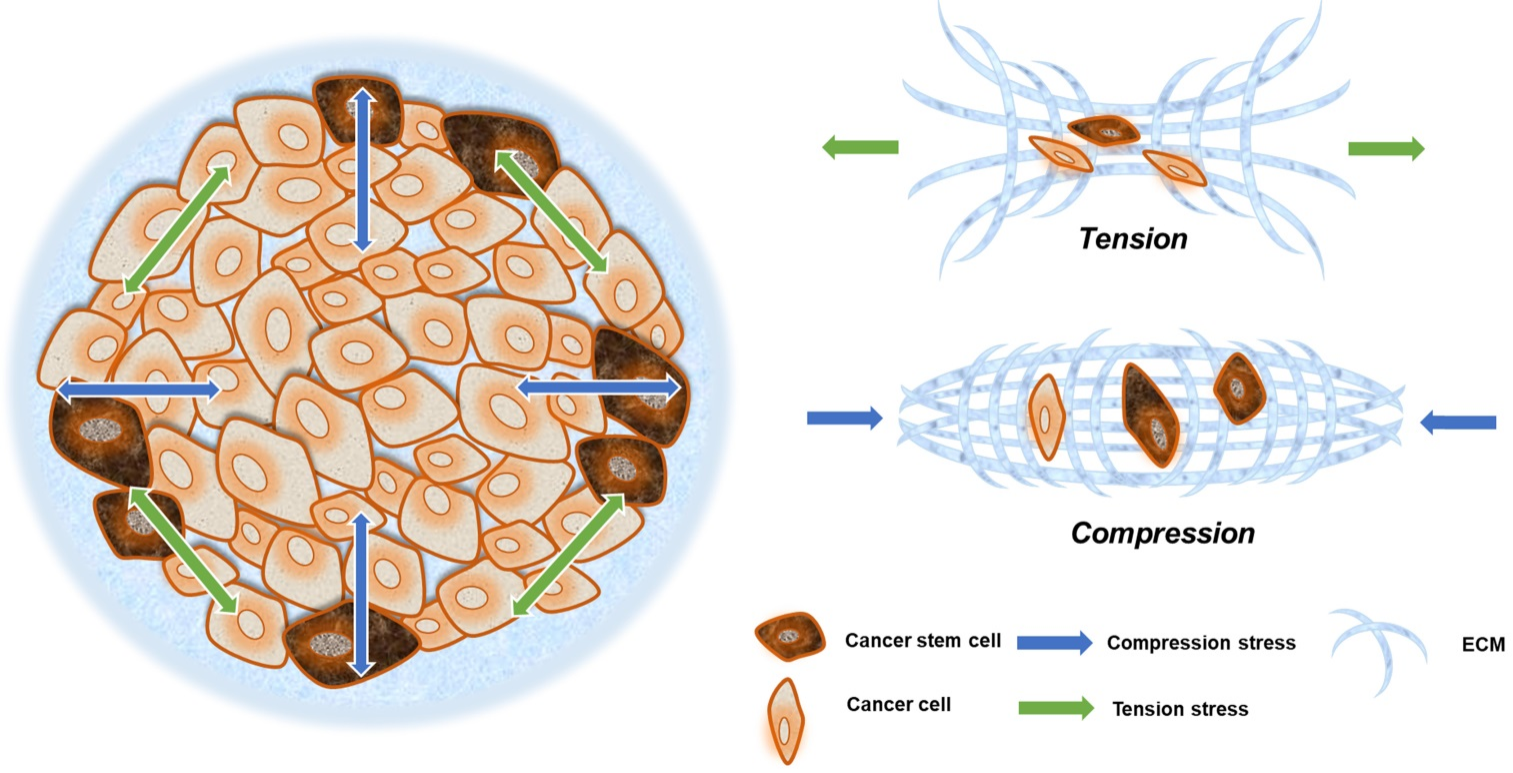
Compression and tension stress in a solid tumor. Neoplastic growth steadily increases tumor size, providing radial tension and axial compression to cancer cells. Cells at the invasive frontier are subjected to tension stress during cancer expansion, and that stress arrives from all directions (green arrow). What is more is that cells at the central area are subjected to compression stress during tissue expansion, and that stress mainly emanates from the invasive frontier directions (blue arrow).

Solid stresses caused by tumor growth can induce a variety of cellular behaviors. Mechanical stresses generated by cells and cellular structures play critical roles in epithelial homeostasis, driving diverse behaviors, such as mammary epithelial cell branching morphogenesis, epithelial-to-mesenchymal transition (EMT), and neoplastic progression 43. The cancer cells in the invasion frontier are subjected to internal tension caused by irregular cancer cell growth, compressive stress from the external ECM, and surrounding paratumor tissue (Fig. 2). This can be thought of as filling a balloon with air, and as the interior expands, the border cells are pressed outward and stretched via interaction with their surroundings.

## Matrix stiffness and CSCs

### Matrix stiffness and the stemness of CSCs

With expansion, abnormal ECM accumulation results in increased stiffness of cancer tissue. Dozens of *in vitro* or *in vivo* experiments have demonstrated that cells are sensitive to reacting the stiffness of the substrate, regulating its migration, differentiation, and proliferation 30. Engler et al. showed that mesenchymal stem cells (MSCs) can differentiate into specialized lineages with various stiffnesses of polyacrylamide gels (PA) gels. The results indicated that MSCs underwent primary neuronal differentiation on soft matrix stiffness (0.1-1 kPa). Stiffer matrices (8-17 kPa) lead MSCs to commit to myoblast differentiation, and a rigid matrix (25-40 kPa) results in osteogenesis 44. The differentiation potential of CSCs can also be regulated by matrix stiffness 45, 46. You et al. showed that hepatoma carcinoma cells presented high stemness on gels with up to 16 kPa stiffness. Furthermore, mechanical signals were transmitted via the intracellular PI3K/Akt-mTOR-Sox2 pathway through the transmembrane protein, integrin-β1 (ITGB1), resulting in high expression of CD133 and epithelial cell adhesion molecule (EpCAM) 47. ITGB1, also known as CD29, is a transmembrane receptor encoded by the *ITGB1* gene in humans. Recent studies have shown that ITGB1 is not only related to mechanical factors but also closely related to CSCs and cancer differentiation 48. Schrader et al. demonstrated that hepatoma stem cells were more abundant on softer gels (1 kPa), a softer matrix can cause hepatoma cells to enter a quiescent and dormant state, increasing their stemness 49. In addition, matrix stiffness significantly affects the stemness of lung cancer cells. The increased matrix stiffness (130-4020 Pa) dramatically elevates integrin-β1 expression for transmitting the mechanical force and further activates the intracellular integrin-linked kinase (ILK)/PI3K/Akt signaling pathway 50. In colon cancer, increased matrix stiffness (2.0-20.0 kPa) inhibits the expression of stemness markers, such as CD133 and acetaldehyde dehydrogenase 1, by inhibiting the activation of yes-associated protein (YAP), PI3K, and Akt through integrin-β1 51. CSCs derived from malignant melanoma have a better ability to maintain stem cell characteristics, exhibiting stronger self-renewal capacity, compared with well-differentiated melanoma cells in a soft matrix 52. Besides, CSCs have low level of histone 3 lysine residue 9 (H3K9) methylation, which is unresponsive to matrix stiffness or applied forces 52.

Although several studies indicated that matrix stiffness significantly affects the differentiation and survival of cancer cells, several researchers have shown contradictory results. Various research models and measurement methods used to assess tissue stiffness may produce varied results in certain tissues. In addition, the selection of different references has led researchers to have varying standards for softness and stiffness. Cancer cells from different tissues have varied sensitivities and adaptabilities to mechanical signals. For example, hepatoma cells show higher stemness in stiff matrix, while melanoma cells express higher stem cell markers in the softer matrix 51-53. To obtain more accurate experimental results, researchers' must be more cautious in the choice of stiffness.

In fact, the cells in the body locate in three-dimensional (3D) environments, and the common methods of constructing different matrix stiffnesses with PA and other gels in the study usually provide two-dimensional (2D) matrix stiffness. By employing the 3D environment to simulate the mechanical environment of cancer cells *in vivo*, the precise information about the actual situation of cancer cells in the body is very necessary. Although some studies have shown that the stiffness of the matrix under 3D conditions affects the stemness of CSCs, the extant literature on the topic compared with the existing 2D conditions is far from sufficient 54.

Matrix stiffness is a key biomechanical force of the tumor microenvironment and correlates tightly with tumor progression. The mechanical receptors on the cell surface, such as integrins, CD44, and ion channels, can sense the change of ECM and activate the downstream key molecules, including FAK, ILK, RhoA, and YAP/TAZ. Some of the signals induce non-CSCs reprograming and transforming into CSCs through highly expressing Sox2, Oct-4 and Nanog 47-53, finally leading to a poor prognosis. Although abnormal matrix stiffness increases cancer cell stemness, there might also be opportunities to take advantage of the abnormal stiffness by developing mechanosensitive treatment. One of the possible therapies is directly changing the matrix stiffness of the tumor by using metformin to reduce the tissue stiffness 55, targeting ECM components such as HA, or changing the secretion of cellular collagenase.

## Fluid Shear and CSCs

### Interstitial fluid pressure in cancer

Interstitial fluid and blood can produce fluid shear stress on cell surfaces under physiological conditions. However, the neovasculature are poorly functional with abnormal vessel network, and have a very high permeability compared to mature microvessels. Within tumors, the increasing number of lymphatic vessels around tumors, which is coupled with high IFP, will lead to an increased tumor fluid flux.

The flow rate of the interstitial flow is slow, and the velocity is just approximately 0.1-2 μm/s, with the resultant fluid shear stress being approximately 0.01-0.2 Pa (0.1-2 dyne/cm^2^) 26. Owing to advances in science and engineering, computational modeling is often used to carry out simulations and auxiliary experiments when evaluating interstitial flow 40, 56. The studies showed that cancer cells had sensitivity to microfluid shear stress *in vitro* and responded accordingly. Dong et al. showed that less fluid shear stress could affect the cytoskeleton and shape cell morphology 57. IFP can increase the migration and invasion of cancer cells by activating CXCR4/CXCL12 and MEK/ERK signaling in hepatocellular carcinoma 58.

The IFP can maintain the biological properties of CSCs. Studies have shown that 0.02 dyne/cm^2^ fluid shear could empower ovarian cancer cells with stronger stemness and EMT properties through microRNA-199a-3p or PI3K/Akt signaling pathways 59. More importantly, the contribution of the interstitial flow is increasing osmolality by causing an increase in IFP 60. IFP can also generate concentration gradients around cancer cells, providing the necessary growth factors and signaling molecules for CSCs 61.

IFP can activate the PI3K/Akt signaling pathway through surface receptors, such as integrins, to enhance the stemness of cancer cells. Additionally, rectifying abnormal IFP due to angiogenesis in the tumor microenvironment is a promising treatment option. For example, acting on VEGFs-VEGFR signaling pathway could reduce angiogenesis to weaken IFP, thereby improving the prognosis of patients 62.

### Blood shear stress and CSCs

Compared to interstitial flow, blood flow has a faster velocity and larger coronary system. The flow of blood can produce greater fluid shear at 1-4 dyne/cm^2^ in narrow vessels and 4-30 dyne/cm^2^ in larger vessels 39. Before entering the peripheral blood circulation, cancer cells exhibit a pronounced dissemination ability after undergoing a series of changes; the biological behavior of cancer cells within the circulatory system is also affected by the action of hemodynamic forces 39. Shear stress is the friction between blood flow and vascular endothelial cells, which regulate cell morphology and function. Fluid shear stress is critical for vascular remodeling and significantly regulates cancer cell metastasis and differentiation 63-65. When enters into the blood circulation system, cancer cells face a completely different mechanical microenvironment 39. Strong fluid shear stress is not suitable for cancer cell survival, although the harsh environment and immune mechanisms in the blood circulation remove most cancer cells, a small fraction of CSC/CTCs still initiate metastasis 66, 67. Several lines of evidence suggest that a presumably small subset of CTCs also bear CSC characteristics based on their ability to give rise to tumors 68. CTCs or CSCs have many similar characteristics, such as a high expression of cancer stem cell markers, CD90, CD44, and EpCAM, enhanced tumorigenic ability, pronounced colony forming ability, and greater EMT capacity 69. Among them, EpCAM is one of the important markers used in clinical screening and identification of CTCs, and is commonly utilized in the study of CSCs and CTCs 70,71. Therefore, several studies in recent years have proposed that the putative source of CTCs is CSCs 72.

Fluid shear stress can affect cancer cell migration through the ROCK signaling pathway, an upstream of the FAK and PI3K/Akt pathways, and finally impact the co-activation of the YAP/TAZ pathway 73. YAP/TAZ, a key transcriptional coactivator in the Hippo pathway, has been reported in many recent studies to be associated with mechanical factors and can respond to a variety of mechanical stimuli 74. Besides affecting the migration and deformation of cells, fluid shear stress promotes EMT in epithelial cells, a process that allow cancer cells to gain CSC phenotype 75, 76. Sun et al. showed that low shear stress inhibited sphere-forming ability, increased chemosensitivity, downregulated CSCs marker expression*,* and suppressed the *in vivo* tumorigenicity of liver CSCs 77. Triantafillu et al. showed that fluid shear stress could promote the expression levels of CD24, EpCAM, Oct-4, Nanog, and other CSC markers, maintaining CSCs within circulatory system in breast cancer cell MCF-7, and this process was independent of EMT 78.

Eliminating CTCs/CSCs in blood stream is always an important strategy for the treatment of metastasis in the clinic. The mechanical signals provide possible treatment strategies for removing CTC/CSCs. For instance, YAP/TAZ might be a new potential target 73. In addition, changing blood shear force may be a new method to eliminate CTCs/CSCs. At present, hemodynamic drugs that have been approved in the clinic, such as cardiovascular drugs and anticoagulants, might also influence tumor metastasis. An improved understanding of how shear stress influences CTC/CSCs at blood vessel could lead to the design of refined anti-metastatic approaches.

## Solid stress and CSCs

Solid stress, as the major component of the biomechanical microenvironment, directly transmits mechanical signals to cells through the ECM, and indirectly affects cells by compressing blood vessels and lymph vessels 79.

Although many studies have shown that tensile and compressive stresses are potential assistors for cancer metastasis, none have directly indicated that CSCs are related to tensile and compressive stress. There is still inadequate explicit evidence to prove that tensile and compressive stress can affect the biological behavior of CSCs. Liang et al. suggested that compression significantly decreased pulposus-derived MSC survival, differentiation, colony formation, and migration. Furthermore, compression loading could downregulate the expression of stem cell-related proteins and result in cell differentiation 80. Gan et al. showed that less compression increased anabolic response, whereas more compression induced the catabolic response of MSCs by inhibiting the expression of transient receptor potential cation channel subfamily V member 4 (TRPV4) 81. Osteocytes also respond to direct compressive stimuli. For instance, the differentiated murine osteoblastic cells MLO-A5, cultured in 3D scaffolds upregulated the expression of osteopontin and osteocalcin when exposed to a compressive loading regime consisting of 5% strain at 1 Hz - 2 h, approximately for three weeks 82. Further investigation is thus needed to determine if mechanotransduction is maintained within cancer tissue when subjected to compression as well as if and how this prevalent mechanical stimulus influences the development of CSCs. Ultimately, research on the CSC mechanical microenvironment can provide more potential theoretical targets for therapeutic intervention of CSCs.

Although the relationship between stress and CSCs needs further research, adjusting the abnormal solid stress may improve response to various treatments, such as immunotherapy. The generation of solid stress is closely related to the ECM composition. Degrading the matrix composition or decreasing the degree of fibrosis will help reduce compression and tension stress. For example, losartan, an antagonist of angiotensin II receptor 1, degrades both collagen I and HA by blocking TGF-β signal 83.

## Perspectives

The mechanical factors play key roles in regulating the characteristics of CSCs. CSCs and normal stem cells often share similar surface markers and signaling pathways, which would restrict the design of treatment regimens 13. In fact, it is challenging for new therapies to target CSCs without affecting normal stem cells. The abnormal biomechanical factors, which rarely exist in the harmonious microenvironment of normal stem cells, may provide new insights for CSC-targeted treatment. As such, discovering the relationship between biomechanical factors and CSCs will greatly enable the generation of novel research strategies to investigate the occurrence, development, and recurrence of cancers.

## Figures and Tables

**Table 1:** Mechanical effects on CSCs

Model	Mechanical environment	Outcome
HCC culture on polyacrylamide gels 58	Substrate stiffness: 6-16 kPa	HCCs show higher stemness on the 16 kPa substrate gels
HCC culture on polyacrylamide gels 49	Substrate stiffness: 1-10 kPa	HCCs show higher stemness on 1 kPa substrate gels
Breast cancer cell culture on polyacrylamide gels and hypoxic environment 50	Substrate stiffness: 0.13-4.02 kPa	Stiffness and hypoxic factors promote the development of breast CSCs
Colorectal cancer culture on Polyacrylamide gels 51	Substrate stiffness: 2-20 kPa	HCT-116 cells show higher numbers of CSC markers with increasing stiffness of gels
Melanoma CSC culture on 3D fibrin gels 52	Gel stiffness: 90-1050 Pa	CSCs have a better ability to maintain stem cell characteristics on a softer matrix stiffness
Ovarian carcinoma cell culture on a poly-HEMA-coated microfluidic channel 59	Shear stress: 0.002-0.02 dyne/cm^2^	Ovarian cancer cells acquired the expression of EMT and CSC markers with 0.02 dyne/cm^2^ shear stress
Breast cancer cell culture in a computational fluid dynamics module 78	Shear stress: 20-60 dyne/cm^2^	MCF7 cells show high numbers of CSC marker under shear stress compared with a static state
Liver CSC culture on a parallel-plated flow chamber system 84	Shear stress: 2 dyne/cm^2^	Liver CSC stemness is reduced under shear stress via the Wnt/β-catenin signalling pathway
Tumor culture on a stress clamp 85	Compressive stress: 5-10 kPa	Tumour sphere volume is reduced under compressive stress
